# Supplementary figures and images for: Effects of root phenotypic changes on the deep rooting of Populus euphratica seedlings under drought stresses
Source: PeerJ. 2019 Feb 28;7:e6513. doi: 10.7717/peerj.6513 (PMC6397757; doi:10.7717/peerj.6513)

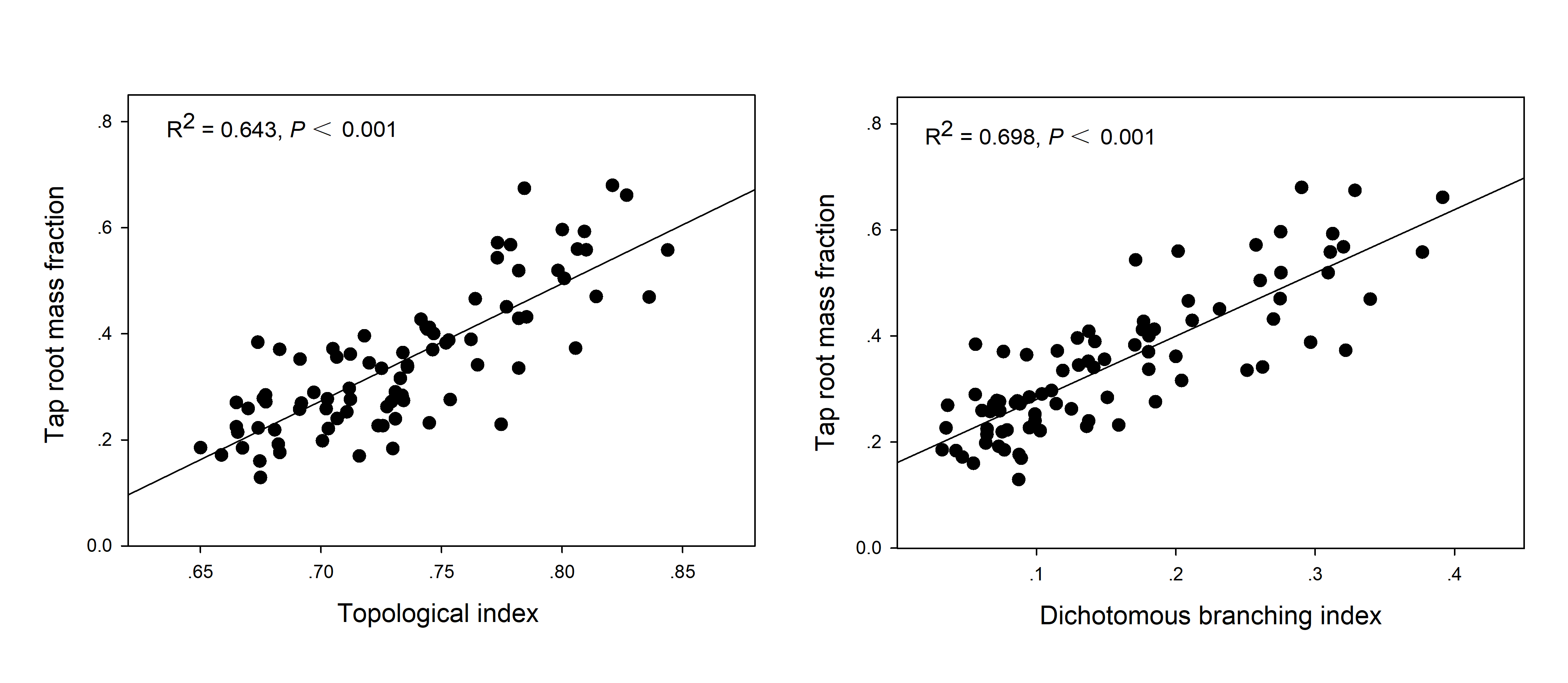

Supplement: Supplemental Information 1 — The correlation between TRMF and commonly used TI and DBI were examined to determine the TRMF availability to representing root architecture in this study. [file peerj-07-6513-s001.png]
